# Supplementary material for: Ecological Relationships Between Woody Species Diversity and Propagation Strategies of Aulonemia queko
Source: Plants (Basel). 2025 Mar 1;14(5):744. doi: 10.3390/plants14050744 (PMC11901908; doi:10.3390/plants14050744)
Supplement: Supplementary file 1 [file plants-14-00744-s001.zip › plants-3469042-supplementary.pdf]

Table S1. Pearson correlations and significance levels (P < 0.05) between response variables related to the spread of *A. queko.*

	Diameter	High	LN	LP	NR	SLA
**Diameter**	1	<0.0001	<0.0001	<0.0001	<0.0001	<0.0001
**High**	0.87	1	<0.0001	<0.0001	<0.0001	<0.0001
**LN**	0.89	0.98	1	<0.0001	<0.0001	<0.0001
**LP**	0.68	0.69	0.69	1	<0.0001	<0.0001
**NR**	0.58	0.59	0.63	0.88	1	0.02
**SLA**	-0.47	-0.55	-0.52	-0.35	-0.28	1

LN: Leaves number

LP: length of the most developed

NR: Number of roots

SLA: Specific leaf area
